# Educational crisis of Rohingya refugee children in Bangladesh: Access, obstacles, and prospects to formal and non-formal education

**DOI:** 10.1016/j.heliyon.2023.e18346

**Published:** 2023-07-15

**Authors:** A. N. M. Zakir Hossain

**Affiliations:** a Bangladesh Agricultural University, Mymensingh, 2202, Bangladesh; b University of Public Service, Budapest, 1083, Hungary

**Keywords:** Rohingya, Refugee children, Formal and non-formal education, Bangladesh

## Abstract

**Purpose:**

The article discusses the contemporary educational crisis of Rohingya children in Bangladesh. It aims to identify the challenges of formal and non-formal education faced by Rohingya children and their prospects. In this scholarship, the author attempts to answer the questions-what are the current formal and non-formal educational services in the Rohingya refugee camps and how and to what extent do the existing educational services intensify the other crisis in their lives, and what are the prospects for them going forwards.

**Method:**

ology: This study is primarily based on secondary sources of data. It focuses on the available educational services and key obstacles that affect Rohingya children's formal and non-formal educational opportunities in Bangladesh and their prospects in Myanmar.

**Findings:**

The study found the absence of formal and non-formal education for Rohingya children in refugee camps has a significant impact on their ability to become an active member of society. Although refugee children and their parents express a willingness to formal education under the Myanmar curriculum, limited educational services and various structural and legal barriers hinder their progress. Finally, the study explores the prospects of formal and non-formal education for Rohingya children in Bangladesh and concludes with recommendations to improve their educational opportunities.

**Limitation:**

The study results can differ in other contexts and countries hosting refugees.

**Practical implications:**

Findings of the study may be helpful for policymakers and practitioners.

## 1 Introduction

The number of refugees in the world has significantly increased [1]. Many countries are facing refugee crises and struggling to provide basic needs, such as shelter, food, and healthcare [2,3]. Refugees are passing through turbulent times and facing numerous challenges worldwide for their life and security [4,5]. Due to the lack of protection and rights in their home countries, many refugees are forced to flee and seek a better life elsewhere, causing a strain on hosting governments in Asia and Africa, who are struggling to provide necessary services and resources [6]. The Rohingya refugees are the most prominent example of displacement in the Pacific Asia region. The Myanmar military's atrocities have forced them to flee their homes, and they have been deprived of their fundamental rights and identity since Myanmar gained independence from the colonial power [7,8]. Millions of Rohingya hosted in Bangladesh started in 1978, and augmented in 2017 and became the highest total displaced in Southeast Asia [9]. Bangladesh currently hosts the largest number of Rohingya refugees, and over half of them are children who require immediate assistance in various areas such as education, health, food, and security [10]. Refugee children face significant obstacles, including limited access to education. Education is crucial for their development and can help reduce inequality [11,12]. UNHCR has reported that a significant number of children are born in refugee camps every year, and without education, they face an increased risk of harm and reduced opportunities for growth and development [13]. Providing education for refugee children is crucial as it can help bridge the gap between them and the rest of the society, leading to better financial opportunities, greater independence, and improved health outcomes. Education can also pave the way for future livelihoods and ensure sustainable development.

The education situation of the thousands of Rohingya children who have sought refuge in Bangladesh, most of whom are not receiving an education, is a matter of concern. The global community needs to take action to address this issue because the future of any civilisation depends on investing in its children and youth. The Rohingya refugee children are in a vulnerable position, and it is crucial to guide them back to the classroom. According to the Reliefweb report.

[14] and UNICEF's estimates, approximately 300,000 refugee children have access to education in 5000 learning centers. Still, about 16% of children aged 3–14 and 81% of adolescents aged 15–24 have no access to education in the refugee camps. Without access to education, children are at risk of being exploited for child labor, trafficking, and sexual abuse [[15], [16], [17]] and extremist ideologies, and parents who cannot find jobs may resort to child labor for income. Further, parents who cannot find jobs and live under legal restrictions may rely on child labour for income in the refugee community, mainly if schools are beyond access.

The concept of education was primarily associated with schools and universities, requiring basic learning skills and leading to a profession. However, over the last fifty years, the concept of education has expanded, and life-long learning has become the central idea in Europe and beyond [18,19]. Consequently, the way we perceived education has evolved and the concept of formal, informal, and non-formal education have emerged. Formal education occurs within a structured system, following pre-defined curricula, and typically involves classes with intended learning outcomes and certifications [20,21]. For refugee children, formal education is important as it provides a pathway for them to acquire knowledge and skills that are recognized by the wider community and can lead to further education or employment opportunities. In contrast, informal education occurs in everyday life, such as community-based training, vocational training, and life skills education. Non-formal education takes place outside of schools and without curricula but under an expert facilitator, it aims to achieve clear learning objectives in different settings, based on principles such as democracy, human rights, and social inclusion [22]. Non-formal education is designed to develop personal and social skills that enable individuals to play a more active role in their community, although there can be overlaps with formal and informal education in the same activity [23]. However, it is often more flexible and adaptable to the needs of specific groups, including refugee children who may have unique challenges in accessing education.

Education is considered a fundamental right of all human beings, crucial for the growth and development of both society and individuals [24]. Non-formal education, on the hand, plays a vital role in enabling active and responsive citizens. It allows individuals to learn from others by asking questions, sharing opinions, and preparing for activities that are essential for refugee children to break free from their existing vulnerabilities. Formal education can be a lifeline for refugee children seeking emancipation from their plight, while non-formal education can train them to become active members of their community or society, allowing them to respond to issues related to their interests. Research has shown that both formal and non-formal education can have a positive impact on the well-being and academic outcome of refugee children. A study conducted in Uganda reported that formal education was associated with increased social integration and reduced levels of psychological distress among refugee children [25]. Meanwhile, non-formal education programs such as vocational training have been identified to enhance the employability of refugee youth [26,27]. In addition, non-formal education that incorporated social and emotional learning activities improves the mental health outcomes of refugee children [28,29].

Refugee children are currently provided only with informal education in Temporary Learning Centers (TLCs) run by NGOs and coordinated by UNICEF. However, both formal and non-formal education equip refugee children with the necessary skills to overcome future challenges and contribute positively to their community or society, providing them with a brighter future and protecting them against child labor, violence, and early marriage. Education can also help in redesigning the civil and political characteristics of refugees in their camps in the host country, making it a transformative tool that is especially urgent for refugee children who are out of normal livelihoods [30,31]. It is widely acknowledged that education can predict a society's civic and political participation. Educational policies and research can positively impact formal education [32,33]. Besides, non-formal education is also crucial for refugee children and youth, especially during a political crisis where the pro-independence attitudes of rulers have complicated their plight. The deeper political interface demands a profound analysis of the role of education which has had a significant impact on the socialization and integration of refugees, empowering them to rebuild their community and society with knowledge and hope. Education is an asset that stays with them wherever they go, and it is the responsibility of the international community to provide refugee children with opportunities to enhance their thoughts, widen their prospects, and support their communities' growth and development. That is why studying formal and non-formal education in the context of refugee children is crucial to identify the barriers to education, improving access to quality education, and informing the development of effective interventions that can improve the well-being and academic outcomes of this vulnerable population.

The main goal of this research is to investigate the present educational crisis faced by Rohingya refugee children in Bangladesh. It aims to analyze the provision of both formal and non-formal education to refugee children in the camps and assess the opportunities and challenges associate with it. The article addresses various issues related to the education of Rohingya refugee children, including the availability of formal and non-formal education, the obstacles they face, and the potential for future education. By examining the current state of formal and non-formal education, and identifying the challenges and prospects, the study provides an understanding of the existing educational framework and opportunities for refugee children, as well as potential obstacles to promoting education and sustainable development.

## 2 Theoretical framework

The arrival of Rohingya refugees in Bangladesh brought with it numerous challenges, similar to those faced by refugees worldwide, as they struggled to adapt to a new and unfamiliar environment where they were not readily accepted into the host culture [34,35]. Around 50% of the Rohingya children are in need of education as a humanitarian response [10], but it is not easy to integrate them into the existing conventional education system, particularly when they have to learn a new language to meet the minimum requirement for interacting and attending school [36]. Therefore, there is a need to find an appropriate teaching methodology to meet the new educational needs of the refugee children. To do so, it is essential to plan for the implementation of formal and non-formal education programs, which should be developed through a framework to ensure harmonized and functional practices. However, the issue of language becomes significant when the GoB refuses to provide education to the refugees in their own language, and there is a shortage of quality teaching staff for both languages. It is also challenging for the children and teachers to learn and teach languages, as it requires a broad understanding of the functions in a multilingual ecosystem when it is connected with social relationships, shaping values, attitudes, and feelings [37]. Literacy in a language is crucial for the refugees to attain various goals, as it empowers them to “read the word” and “to read the world” to learn new knowledge and develop their skills [38]. Formal education is essential for all, while non-formal education is an integral part of education in general, involving a “deliberate, systematic, sustained effort to transmit, evoke or acquire knowledge, attitudes, values and skills or sensibilities” [39]. The distinction between formal and non-formal education lies not in their aims and functions, but in the variety of fundamental key behaviors that lead to a legal and valid behavioral pattern [40,41]. Furthermore, non-formal education can adapt to changing environments and educational challenges with its inherent flexibility, particularly when the challenges are pertinent to the psychological development of younger children.

The theoretical underpinnings of formal and non-formal education for refugee children are essential for their educational development, socialization, and future prospects. One of the theoretical underpinnings of this study on formal and non-formal education for refugee children is the concept of education as a human right. The Universal Declaration of Human Rights, adopted by the United Nations in 1948, recognizes education as the fundamental right. This principle is reinforced by various international laws and agreements, such as the Convention on the Rights of the Child (CRC), which emphasizes the right of every child to education [42,43]. Another important framework is culturally responsive education, which aims to create an inclusive learning environment that recognizes the learners' cultural background and diversity, particularly relevant for refugee children who come from diverse cultural backgrounds and may have experienced trauma and displacement [44]. Additionally, the socio-cultural theory of learning highlights the significance of social interactions and cultural contexts in shaping learners' knowledge and skills, emphasizing the importance of creating a supportive learning environment that provides opportunities for social interaction and collaborative learning in both formal and non-formal educational settings [45,46]. In addition, integration theory suggests that refugee children should be integrated into the host country's education system to reduce marginalization and promote social cohesion. This theory advocates for formal education within the host country and for non-formal education programs to supplement formal education, as studies found that, both have had importance in their development [47].

The theoretical approaches suggest that children in crisis or emergencies need to be integrated into the education system for their legitimacy and personal identity through formal and non-formal education, enabling them to adapt to a multicultural environment. For refugees, their experiences of displacement and rebuilding a sense of belonging in a new country can be challenging. Therefore, creating an atmosphere of respect and recognition through education is essential to help them reconstruct their sense of belonging, which is crucial for restoring their confidence and belief in future prospects through life-long learning.

## 3 Research methodology

The study focuses on analyzing the educational crisis faced by the Rohingya refugee children, using mainly secondary data sources. Rohingya refugees have been displaced and deprived of basic services since decolonisation, making them the most displaced people in Southeast Asia. The objective of the study is to assess the pros and cons of current formal and non-formal education provided to Rohingya refugee children in Bangladesh, as well as identify future opportunities. The study primarily relies on academic and grey literature as data sources. While grey literature may have limitations in terms of authorship, bibliographic standards, reliability, and authenticity, it can still provide valuable information that traditional publications and databases may not include [48]. The author took care to consider only reliable and credible grey literature for this study, despite the potential limitations.

The study concentrated on using secondary sources specifically related to Rohingya refugees and their children. The author followed a systematic approach [49] to gather data from different databases such as Google Scholar, Research Gate, Mendeley, Scopus, ProQuest, and Web of Science. The author used keywords such as ‘Rohingya’, ‘Refugee’, ‘Rohingya Refugee’, ‘Refugee Children’, ‘Children’, ‘Education’, and ‘Bangladesh’ to find literature related to the educational crisis of Rohingya refugees in Bangladesh and Myanmar. The author included both primary and secondary studies, such as journal articles and reports from various international organizations written in English. The study aimed to synthesize previous research on funding for advancement and overcome the limitations of individual studies focusing on only one aspect of refugee children's education. The study focused on formal and non-formal education facilities, existing challenges, and future opportunities for education for Rohingya refugee children in Bangladesh.

## 4 Literature review on Rohingya refuge children in Bangladesh

**Fig. 1:**
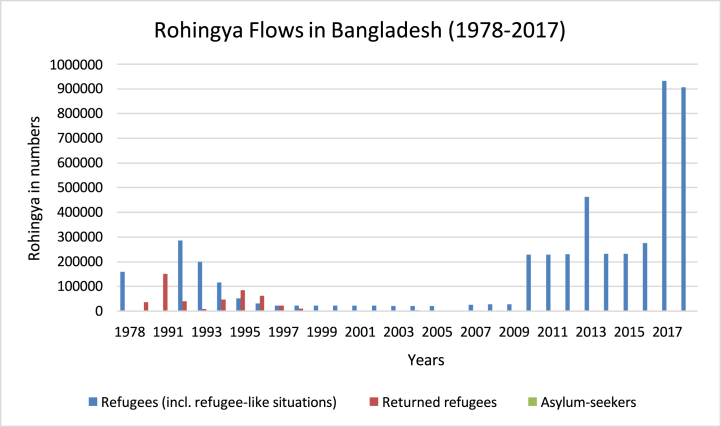
Rohingya refugee flows in Bangladesh (UNFPA, December 2019).

The Rohingya community, who have been displaced since the decolonisation era, is considered the most vulnerable and disadvantaged community in Southeast Asia, lacking rights and opportunities. They began fleeing from Myanmar to Bangladesh in 1978 and their situation worsen in 2017 when the military government of Myanmar started killing them and burning their houses in Rakhine (Fig. 1) [50]. Myanmar was established on a weak structural base by its colonial power, and ethnic groups were used to consolidate its rule, leading to the exclusion of the Rohingya from national resources for over fifty years [51]. As Johnson [52] noted, “the colonising nations affected the history of political ideologies and policies to set up standard social and economic policies for the colonised nations."

The emerging field of nonfiction literature in refugee studies mainly focuses on refugees' experiences, starting from their involuntary displacement, especially when it involves ethnic cleansing and the study of genocide [53,54]. However, in 2017 Rohingya crisis was not the first instances of violence against this group, as similar incidents occurred in the early 1990s [55]. The Rohingya community has suffered a great deal due to the policies of the state's ruling minority, including military dictators' establishment of “*disciplined democracy*” to restore order [56]. This has resulted in the displacement of thousands of people, including Rohingya, in Rakhine. The global literature reveals that refugees and displaced persons are the consequences of war, conflicts, natural disasters, and political unrest, which has led to the development of international non-state players' functioning structure (i.e., UN, UNHCR, IOM, WHO, UNICEF, etc.) from relief to coordinate their humanitarian development activities [57] and categorising the transnational laws to protect the refugees' rights [58]. As a result, refugees' rights and opportunities vary depending on the domestic laws of the host country, and many countries are out of the 1951 refugee conventions and its protocol of 1967, which allows them to limit refugee rights. Thus, the focus is on the emerging refugee field that combines refugee experiences from composition to migration and humanitarian responses from the aid agencies and host countries to illustrate their experiences that can lead to their future well-being [59,60].

The Rohingya refugees are the most displaced and live in overcrowded refugee camps, facing constant adversity. The Myanmar government is not supportive of creating suitable conditions for their voluntary return, and efforts of regional and international nations and humanitarian organizations have been unsuccessful in convincing them otherwise. The international community has not been effective in addressing this crisis, and the interests of regional powers have hindered the refugees' peaceful settlement [61,62]. Resolving this requires collaborative, and global initiatives that not only focus on the movement of refugees but also their past and the issues of national security, border control, identity, citizenship, and statelessness. The role of local and international agencies is to provide assistance, establish, and manage camps and facilitate resettlement [63], while ensuring the protection and advancement of refugees’ rights and opportunities.

**Fig. 2:**
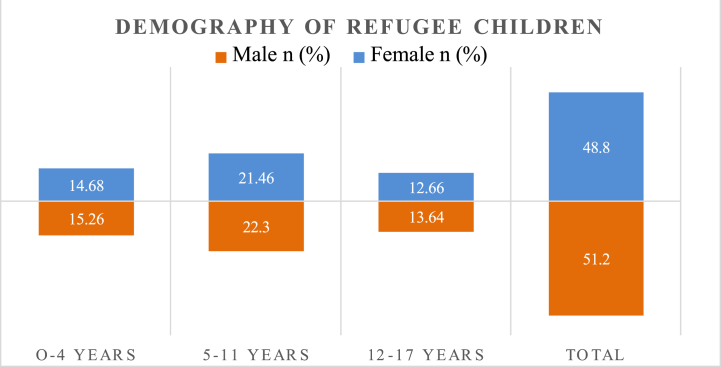
Demography of refugee children in Bangladesh [65].

During the late twentieth century, only 300,000 refugees sought shelter in Bangladesh, due to the socio-political turmoil in Myanmar, this number has increased significantly in the second decade of the twenty-first century. In 2017, more than 700,000 refugees fled from military brutality in Rakhine, with 90% percent of them seeking refuge in Bangladesh. Numerous studies have shown that women and children are especially vulnerable during civil war or conflict, and this is no exception for the Rohingya, as evident in Table 1. A concerning aspect is that more than 50% of the refugees are children who require basic necessities such as food, education, and healthcare to survive. Although educational support is critical, it is often inadequate, with a greater focus on food and health. Nevertheless, providing robust educational support can benefit both refugees and the host society. Over the half of Rohingya refugee population consists of children, of which 70% are adolescents (Fig. 2). These adolescents have experienced stress and trauma and require care to ensure their future well-being. In their distressed journey, refugee children need special support to learn, develop, and create integration and repatriation possibilities [64]. The previous Rakhine incidents have had a severe impact on the mental health of these children, which earnestly requires proper intervention, and education can be a lifeline for their recovery.

**Table 1:** Demographic distribution of Rohingya refugee.

Age Group	Male	Female	Total
	n (%)	n (%)	n (%)
Infant-17 years	26	25	51
18–59	20	25	45
60+	2	2	4
Total	48	52	100

## 5 Findings on formal and non-formal education for Rohingya children

The response to the Rohingya crisis has not yet ensured the community's dignity and self-reliance, but education is inevitable for any group's sustainable development in society. Additionally, skill-building and education are vital for effectively managing refugees and achieving practical, long-lasting solutions. The number of refugee children in the Bangladesh camps is around 400,000, and it is the responsibility of the host country to provide educational facilities for their future well-being [66], as recommended by international law for refugees (Article 22) that mandates equal opportunities for refugee and local children [67]. Education is also essential for improving refugee livelihoods and increasing their opportunities to migrate, repatriate or reintegrate.

### 5.1 Existing formal and non-formal education: structure, logistics, and support

#### 5.1.1 Structural and logistic support for refugee children's education

The main issue concerning education for Rohingya children is the government's policy, which denies them formal education due to their legal status and documentation [68,69]. The policy prevents around 400,000 refugee children from accessing education, leaving them without the opportunity to receive primary and secondary education in their camps. The government is resistant to providing permanent structures in the camps, as they anticipate the refugees' return to Myanmar when conditions improve [70]. However, the government acknowledges that education is essential to empower refugee children and reduce their dependence on the host country for enduring harmony and societal cohesion. Despite this, the government has only allowed informal education for Rohingya refugee children in English or Burmese in the refugee camps and is unwilling to provide financial support [70]. As a response to this, NGOs has established temporary learning centers that can accommodate only a small number of students [71]. These centers have no tables, desks, or electricity, and children receive only 2 h of schooling due to a shortage of space. Religious schools (Quami Madrasa) are also available in the camps, but they are not recognized by the education system of Bangladesh. In addition, multidimensional barriers, including socio-cultural factors, economy, safety, hygiene, and toilet facilities contributed to limited access to education, particularly for females [10].

**Fig. 3:**
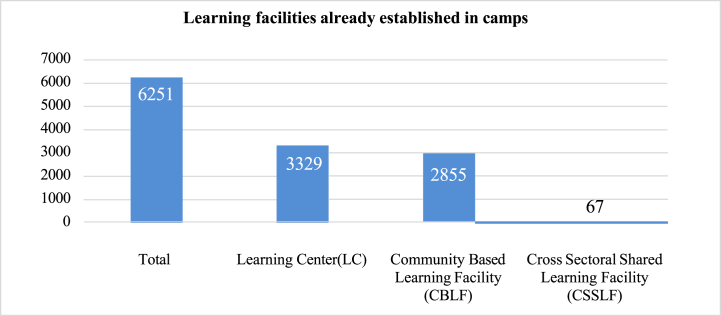
Types and numbers of (informal) learning centers [71].

In 2019, around 3200 learning centers were operational, and 70% of them were supported by UNICEF. Currently, there are over six thousand learning facilities (as shown in Fig. 3) in the refugee camps, which includes a learning centre (LC), a community-based learning facility (CBLF), and a cross-sectoral shared learning facility (CSSLF). However, despite these efforts, there is still much to be done to improve the situation of refugees, as only 30% of the Joint Response Plan (JRP) targets have been achieved in the camps [14]. Consequently, due to legal and administrative barriers and a lack of quality teaching staff, educational preparedness is insufficient for children and youth in the Rohingya camps.

#### 5.1.2 Legal framework for education to Rohingya refugee

The safety and security of individuals are no longer solely the responsibility of a single state, as international law has been recognized the rights of minorities, whether they are defined by-ethnicity, religious, or linguistics. Rohingya refugees in Bangladesh have been issued identity cards by the Ministry of Home Affairs to improve service delivery and protect them, but they still lack of formal education and economic integration, as well as other basic rights. Despite being neither a signatory to the 1951 Refugee Convention nor its 1967 protocol, Bangladesh has failed to provide refugees with formal education and economic inclusion, instead offering vague commitments for their future. While several international laws protect the rights of refugees, Bangladesh has made a reservation about some of them such as the Convention on the Elimination of All Forms of Discrimination Against Women (CEDAW), Convention on the Rights of Persons with Disabilities (CRPD), and two of Convention on the Rights of the Children (CRC), when ratifying their optional protocols. On the other hand, the Global Compact on Refugees (GCR) 2018, an intergovernmental treaty, emphasizes the rights of refugees, including their right to education, in paragraphs 68 and 69 [72] building on International Human Rights Law and the Universal Declaration of Human Rights and expanding it in the CRC. CRC seeks to legally replicates the status and rights of refugees, promoting non-regression and non-discrimination, and serves as an instrument for cooperation between multiple countries and other non-state actors [73]. GCR empowers both states and the United Nations to protect the rights of migrants and refugees [74], with a particular focus on education for the refugees and sustainable development.

#### 5.1.3 Teachers and teaching facility at camps

In order to educate refugee children, it is crucial to have well-trained and properly equipped teachers in emergency education. This requires attention and support at both the policy and operational levels. Teachers play a significant role in transferring knowledge, supporting children's growth, promoting their potential, and helping them navigate their future. There are handful numbers of university graduates available in the Rohingya refugee camps to serve as teachers. Donors and humanitarian agencies are suffering to recruit enough instructors with at least a high school education to operate the informal learning centers. A recent report on Rohingya education reveals that when selecting teachers, academic qualifications, experience in teaching, English language proficiency, and age are prioritised selecting teachers among the host society, while, educational qualifications, age, the ability to conduct lessons, and educational certification are prioritised for Rohingya [75]. Trained and skilled teachers are essential to achieving educational goals for refugee children. Teachers have the ability to inspire and learn from children and guide their intelligence, and they are the most important factor in providing quality education, even more so than the physical school infrastructure. Therefore, well-trained and skilled teachers are needed for both formal and non-formal education in refugee camps. Pre and in-service training is critical for teachers, as it can improve their employment prospects and encourage to deliver better services for refugee children. However, the existing informal education system has limited potential for achieving the structural benefits of education due to the lack of training, resources, and logistics for the teachers. While their performance can be improved by developing skills to promote formal and non-formal education, the teachers receive little training on informal education [71] and no expert orientation for non-formal education within the existing framework.

#### 5.1.4 Existing educational structure in the refugee camps

Refugee children are receiving education through the “Learning Competency Framework and Approach” (LCFA) an informal education structure adopted and implemented by UNICEF. The majority of the children are at LCFA levels one and two, which corresponds to primary level two in the formal education structure. The LCFA covers only four subjects (English, Mathematics, Burmese, and Life Skills) and teachers are from the Bangladeshi community, with the assistance of Rohingya refugee in teaching Burmese. Although this has improved the situation of refugee children, the lack of curricula, structured lesson plans, textbooks, and inadequate training has resulted in lower educational quality. In addition, government policies have prevented refugee children from pursuing formal education and taking national examinations [70]. Besides, the absence of plans and programs of non-formal education hinders the development of skills and competencies. UNICEF provides informal education to adolescents, and this is insufficient for those aged 15 to 18 who need a high school education to prepare for the undergraduate level. According to the UNHCR report [76], there are structural barriers to refugee students accessing post-primary education, resulting in significant enrolment inequalities at all levels of education. To address this issue, Caritas Switzerland has developed a pedagogical approach called the ‘Essence of Learning’ (EoL), which offers mental and educational support during crises and includes weekly monitoring of educators and sensitisation workshops for parents and caregivers [77]. This program represents a small step toward overcoming the challenges faced by refugee children and improving their prospects for the future.

### 5.2 Challenges of formal and non-formal education for Rohingya

#### 5.2.1 Absence of structured curriculum for formal and plans for non-formal education

The future of Rohingya refugee children's education is uncertain due to lack of a structured curriculum for formal education. Curriculum development is essential for early education, as it helps create interest among students and enables the transfer of acquired knowledge. In International and Comparative Education (ICE), curriculum development is the fundamental aspect that focuses on teaching, funding, and management methods based on the political changes, and labour market trends. However, the absence of a structured curriculum has resulted in only informal education being available to refugee children aged four to ten, with no formal education or non-formal education programs for the older age group. Non-formal education is an alternative and supplement to formal education, that is organised outside of schools for developing work and life skills for social and cultural development [78]. The benefits of non-formal education are numerous-including flexibility and adaptability to society's changing needs and attitudes that can foster tolerance, a valuable asset for refugee children. Additionally, language is crucial for their formal education.

#### 5.2.2 Language barrier

In 2017, the government of Bangladesh banned education in the Bengali medium to prevent demographic integration, causing learning centers to stop offering education in Bengali. This ban has affected many children who were born and raised in Bangladesh and have never been in Rakhine. The government suggested redesigning the curriculum for all Rohingya for their further education in the Burmese language [79], but in refugee camps, students only receive informal instruction in English and Burmese. This is because, many educators and instructors are from Bangladesh and are not proficient in Burmese causing problems in the education process. Moreover, Rohingya speak a local language, that does not have a written form and use Bengali keywords to communicate with aid and development workers in the camps. The effectiveness of monolingual and bilingual instruction is currently unknown. The language mix in the Rohingya refugee camps has turned into a political battleground and a source of integration and exclusion from both Bangladesh and Myanmar. The absence of formal and non-formal education, along with language barriers, has exacerbated their crises faced by Rohingya children, impacting their future well-being and gender-based equality at the refugee camps.

#### 5.2.3 Absence of gender equity in education at refugee camps

Incorporating both genders in mainstream formal and non-formal education is necessary for holistic development of refugees and the host society. Educating mothers, in particular, is widely recognized to have a positive impact on health and education of their families, including reducing rates of early marriage, unwanted pregnancy, and infant and maternal death. However, refugee girls face challenges in accessing education, with fewer girls than boys attending primary school globally and even fewer attending secondary school. Educated mothers can also care about safe and clean water and healthy food for the children and hygiene issues of their surroundings, which is crucial to protect their family members from threats. Refugee girls, in particular, face discrimination, cultural conflicts, and mental and physical harassment, which demotivates them from attending school, especially during young adulthood [80]. Limited participation of adolescent girls in Rohingya camps further exacerbates these challenges, with an overcrowded camp lacking suitable space for teaching and studying [75]. The current informal education cannot provide gender equity teachings, which is where formal and non-formal education can make a difference. Structured curriculums and planned systems can provide the opportunity for girls to learn about their equal rights and potential, leading to comprehensive and sustainable development where girls are treated equally in society.

#### 5.2.4 Certification and future higher education

The education sector reports [81] that registered refugee children who receive an education can obtain a certificate upon completion; however, children who arrived during the 2017 influx are not allowed to receive certificates [37]. Some children were previously able to receive education through the local community, and a few even obtained undergraduate degrees using false national identity cards. The newly arrived children are hopeful about receiving formal education at the secondary and higher levels in Bangladesh, which would support them in the future [80]. Non-formal education at these levels can offer refugee children with a better understanding of their surroundings and enhance their skills and potential, making them more responsible members of their society who can respond better during a crisis. However, expert and trained teachers are required and continuous training is necessary for ongoing benefits.

#### 5.2.5 Inadequate number of teachers and training

The proportion of teachers in refugee camps is less than one per thirty-five students for Rohingya refugee children. This inadequate number of teachers with limited skills has led to poor learning outcomes in the refugee camps at Cox's Bazar. Due to their traumatic situation, refugee children require extra attention from their teachers. Without proper training and appropriate skills, teachers may not understand the students' mental abilities and fail to achieve their teaching goals. Both pre and in-service training are essential for newly recruited and experienced teachers for both formal and non-formal education and intended learning outcomes, but such training is limited in the refugee camps.

#### 5.2.6 Proximity and polarisation of religion-based education

The impact of religious education has been significant in the life of Rohingya both in Myanmar and Bangladesh. The Prime Minister of Bangladesh allowed the opening of Quami Madrasa in refugee camps and gained popularity as a solid alternative to formal education, especially when it is not available [82]. Many Rohingya children prefer Islamic education over informal learning centers, and their parents also prefer to send them to the madrasa [37]. However, the perceptions of both students and parents about the learning center is not favorable and hindering the development of their skills. For example, a girl answered a question about the learning center that “*it was serious, while the learning centers are for playing, not for education*” [70]. Consequently, there is a smaller participation of adolescent girls in learning centers compared to boys because the parents do not want to send them during puberty [37,80]. Unless the perception of the students and guardians changes, development through formal and non-formal education will not be possible. Furthermore, studies suggest that non-formal religious education is incapable of equipping individual with new skills and techniques necessary for professional development and being responsible citizens [83]. Both teachers and students in Islamic study centers face numerous challenges in terms of funding, services, and organisational fragility [84].

#### 5.2.7 Prone to natural disasters and unhealthy environment

The refugee camps in southern Bangladesh are susceptible to natural disasters, particularly during the monsoon season [85]. This region is prone to landslides during this season, and inadequate sewage and sub-standard toilets pose a danger of overflow. The pungent smell from the overflowing sewage makes it challenging for children and teachers to attend the classes. In addition, ear-splitting intercepted the class during the construction works around the refugee camps, while the loud noise from construction works around the refugee camps also hinders their studies. Furthermore, learning centers are poorly built, making them vulnerable to damage during heavy rains and storms. These learning centers are not as well-established as formal schools in the host community. Due to the restricted mobility of refugee children and youth, non-formal education is crucial for them within the existing administrative framework. Nonetheless, non-formal education can be beneficial for the children in times of crisis and can help them support each for better livelihoods within the refugee camps.

#### 5.2.8 Less time and involvement in education in and outside of the centers

The limited classroom space and high student population in the learning centers make it impossible to have more than 40 students in one class, resulting in shorter class times and multiple shifts in the same place. In addition, informal education does not motivate Rohingya children as does not offer any prospects future studies, despite parents being interested in income-generating activities for their children, which have limited opportunities. Moreover, instructors struggle to manage official formalities and meetings with various NGOs and GOs, and other humanitarian agencies, reducing the interest and time spent in the informal learning centers. In contrast, formal and non-formal education can change the mindset of both the children and parents, increasing their interest and enthusiasm as it provides prospects for their career and personal development.

#### 5.2.9 Power conflict among teachers and superior complexity

The Rohingya refugee faces multiple vulnerabilities, including insufficient number of teachers recruited to teach their children. Prodip and Garnett [37] found a ‘cold conflict’ between the Bengali and Burmese teachers. The Bengali teachers always feel superior complexity and try to control the Burmese teachers and are forced to follow their instructions as they are vulnerable. Also, Bengali teachers are comparatively more educated than Rohingya teachers, inspiring them to look at the Burmese teachers with a downgraded outlook. This mind game created an unpleasant situation among the teachers and impacted the refugee children's informal studies. A formal education system can reduce this power conflict. It has a standard and legal framework for recruitment irrespective of caste, religion, and ethnic groups that can allow them to enjoy equal rights and benefits for the terms and conditions of their positions. Moreover, positive teacher relationships can impact both formal and non-formal education outcomes for the students at all levels, increasing prospects for success.

### 5.3 Prospects of formal and non-formal education

#### 5.3.1 Rohingya parents and children's readiness for formal education

The Rohingya refugee children and their parents are eager to resume formal education. The country director of Bangladesh's Save the Children also reemphasised their educational emergency. He said, “e*fforts need to be re-doubled to provide quality education to Rohingya children. This can be achieved through community outreach to convince families to send their children back to school and by urgently resuming the rollout and expansion of the pilot program to allow Rohingya children to study in their mother tongue using the curriculum.*” However, due to lack of logistics and trained teachers, non-formal education is not currently available in the camps where the children are confined. The absence of trained teachers and facilitators to conduct the lesson and session made it beyond imagination. Non-formal education is crucial because “*every individual has to learn and practice what they want to achieve perfection*” as the great philosopher Socrates mentioned. It focuses on the process and can also change and influences one's knowledge, skills, and attitudes. It can also broaden the curious mind and stimulate searching for answers and self-esteem to obtain value with formal education. Nowadays, non-formal education is given priority in youth development to help individuals learn consciously and map their skills and knowledge to become members of their community and to complement formal education.

#### 5.3.2 Recent development on the formal curriculum and certification of education

The learning facilities available to Rohingya children need to be improved. Despite a large number of teenagers aged 15 to 18 not having access to education, only a small percentage of refugee children are currently attending school [86]. In response to the urgent need for education among the refugee population, the Government of Bangladesh (GoB) has authorized the use of the Myanmar educational system, and local and international humanitarian partners have taken the responsibility for educating Rohingya children in Bangladesh. The initial phase targets 10,000 refugee children, grades six to nine [87]. The Rohingya people see participation in formal education under the Myanmar educational system as a crucial step toward their eventual return to Myanmar and reintegration [88]. To date, this is the latest initiative to address the formal education of refugee children. They can get the certificate for their education and use it anywhere in the future. “In emergency context through the recovery, it is important that national authorities, educational institutions, and employers recognise curricula and the certificate awarded. Communities want to that their children's education has a value that national authorities recognise that value” [89]. This initiative has a significant impact on the refugee community, Taslim, a nine years old student, reported, “*this would help me to be a doctor or teacher and be able to fulfill my dreams*,” when talking with Save the Children, co-leader of the education coordinator at refugee camps. It is also true that offering non-formal education with formal education could help them build up their future with hundreds of possibilities within their earned potentials that society requires nowadays.

#### 5.3.3 Formal education preparation and future expansion

As mentioned earlier, the GoB has approved a pilot program to provide formal education to ten thousand Rohingya children from sixth to ninth grade who have had limited educational opportunities than their younger counterparts. This initiative will require an additional 250 teachers, with the existing 8900 teachers. Since refugees start their formal education under the regular curriculum, they will become more confident about their future. Then refugee people also get equal employability in any society if they fulfil the recruitment criteria. If and when safe and dignified circumstances allow for voluntary repatriation, education under the formal and familial curriculum will support their future integration into the education system of Myanmar. Furthermore, humanitarian agencies announced their plans to expand formal and non-formal programs to increase the capabilities of refugee children, contributing to their future development. However, it requires an institutional and organisational structure to provide formal education to them.

#### 5.3.4 Structural development for refugee education

Infrastructure development is underway to improve the informal education of refugee children. Recently, several sub-sectors have recognized the importance of implementing formal primary and secondary education in Rohingya refugee camps. New buildings, furniture, and wash block have already been made to intensify the informal primary education for many refugee children. Although the playground is partially constructed, a solar panel for continuous energy supply has yet to be established. Additionally, a multimedia classroom has been established for 60 primary-level students. The government of Bangladesh initiated scholarships for meritorious students at the secondary level and improved logistics including classrooms, benches, multimedia facilities, science labs, and wash blocks have been established with the help of the Japan International Cooperation Agency (JICA) and GoB to expand the formal education in the refugee camps. To complement this formal education, it is necessary to structure non-formal education for the refugee children and youth as part of their lifelong learning process in various settings and improve teachers' training.

#### 5.3.5 Training for the teachers

Refugee children have specific needs and vulnerabilities that require teachers who are skilled and trained to understand their particular needs. Both the newly recruited and experienced teaching staff need cross-cultural training to conceptualise and encounter the needs of helpless children in the refugee context [47,90]. The education sector of Bangladesh has been shut off since March 2020 due to the Covid-19 outbreak and resumed slowly with continuous mass vaccination in later 2022. Therefore, a new way to educate Rohingya children is essential, where ICT intervention is the best and most suitable alternative for continuing informal education in this crisis period. The government of Bangladesh has trained teachers to cooperate with other stakeholders to promote ICT-based learning for refugee children. Foundation training, E−monitoring, and distance learning program orientation have allowed teachers to share their knowledge among themselves and with students, building more interactive connectivity. An increasing number of sub-cluster training will help the teachers concentrate more on their students for achieving their education goals. In addition, the latest recruitment of teachers will reduce the teachers' load and help to contribute more focus on students' learning outcomes. To make it happen, the teachers need professional development through a structured framework for continuous improvement of knowledge and skills [91]. Besides, the training on ICT-enabled learning and sub-cluster training will strengthen teachers' capacity to develop more interactive lesson plans, deliver lessons for formal education and construct non-formal education plans and programs for better lifelong learning outcomes.

## 6 Discussion

**Fig. 4:**
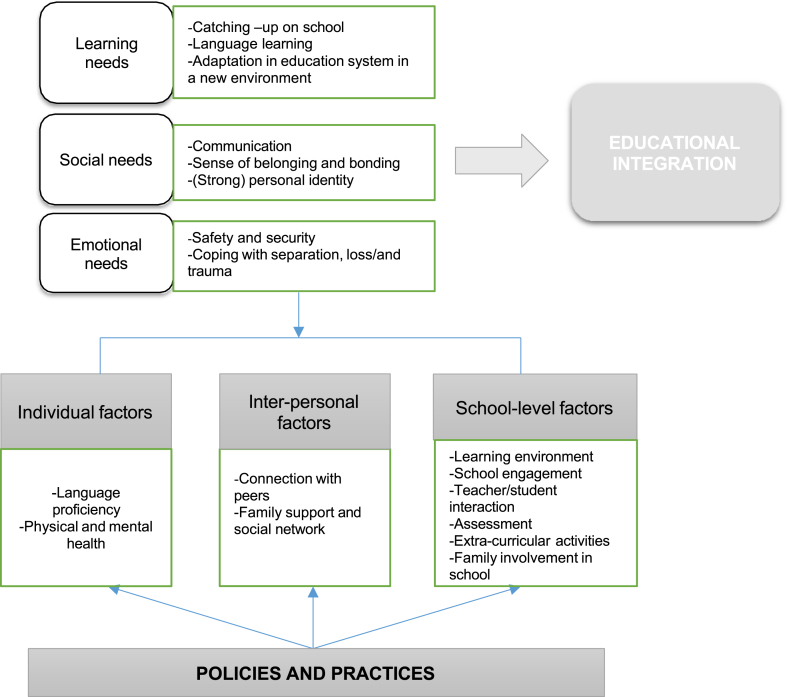
Inclusive model for educational integration of refugee children [47].

This study examines the educational crisis of Rohingya refugee children in Bangladesh, particularly in terms of formal and non-formal education available at their camps. In order to address the needs of these children, it is important to identify the challenges and prospects of the existing educational framework. Refugee children face a myriad of challenges in their camps, and addressing their specific needs is crucial for their integration into an education system (Fig. 4). By providing formal and non-formal education, their sense of belonging, identity, and protection can be strengthened, helping them overcome trauma. A structured curriculum is a prerequisite for the successful integration of refugee children into formal education, providing them with comprehensive development opportunities in the camps. Inclusive education models that consider their needs, policies, and partnering with allied agencies can be used to address these needs (Fig. 4). The study found that refugee parents and children are eager to learn under formal curricula which enlighten them to look at the world around them and respond. The language of instruction, Burmese, is an asset to them in their educational journey. Non-formal education can help refugee children gain civic and political knowledge, which can be useful in their camp life and even after repatriation or resettlement. However, non-formal Islamic education (NFIE) in Rohingya camps lacks essential components such as questioning, interactions, and debating which are crucial for critical thinking-a core skill that can be fused with formal education [83]. Integrating refugee children into education acknowledges their diversity and capability to navigate multiple cultures [92]. Inadequate knowledge of language and discouraging teacher approaches are the main challenges in non-formal education for critical thinking in Rohingyacamps. Despite these challenges, teachers can create a “*creative alternative space of becoming*”[93] for children, helping them renegotiate the borders of identity and belonging. They can build an environment “*conducive to creating futures, rather than simply inheriting them*” [94]. This study also acknowledges that Rohingya children require specialized assistance in various forms to reconstruct and rebuild their selves, allowing them to move forward with the support of education.

Regarding the education of Rohingya refugee children, teaching them in the host language is challenging due to government policy, while teaching them in their original language is hindered by several factors such as the uncertainty of repatriation and inadequate availability of teaching staff. Regardless of the language used for instruction, creating an environment that recognizes their abilities and potential is crucial for the educational development of enthusiastic refugee children. By using their original language, education can focus on fundamental communication skills and engage them in innovative collaborations for daily activities, even with limited competencies. Such an approach can provide a therapeutic environment for traumatized children and help rebuild their sense of belonging and confidence [95].

The social inequalities of the Rohingya people and the crisis in the refugee camps have worsened the pre-existing disparities in formal and non-formal education opportunities. In order to address these issues, it is essential to implement community-based programs that focus on reducing gender-based gaps in education in the camps. Bakali and Wasty [96] recommended the establishment of Islamic education centers as a means of achieving this goal. Regarding the language of instruction, Rohingya parents and children have opted for the Burmese language due to their basic knowledge of it, while the Bangladeshi government does not permit them to learn in Bengali. However, language preference can also be influenced by social prestige [97]. While education in Burmese may facilitate repatriation, it is essential for the government of Bangladesh to recruit and teachers who can teach the Burmese language. According to UNICEF spokesperson Karen Reidy, “*the advantage of learning Burmese is that it would provide an opportunity for Rohingya children to reintegrate into the education system in Myanmar in the future. It is their national language*.” However, excluding the Bengali language could result in their marginalization in the host society. It is noteworthy that formal and non-formal education have limited potential scope to yield positive outcomes in the present situation due to the inadequate content in the existing educational structure. On the other hand, Syrian refugee people in Turkey are allowed to join the national curriculum and benefit from language development, free movement, job opportunities, and social cohesion strategies [98].

This study emphasizes the importance of education in emergencies (EiE) for the Rohingya community [10], but highlights that it may not be enough due to the uncertain nature of their repatriation and prolonged statelessness. Without host country's willingness and repatriation efforts, there is a risk of a ‘lost generation’ in the Cox's Bazar refugee camps. The initial ‘interdependent’ migratory flow with eventual repatriation may transform into a James Rosenau ‘turbulent’ and anomic situation in the absence of appropriate support and intervention [99].

Refugee children have the potential to enhance their non-formal education with relevant resources, even amidst crises such as covid-19 pandemic and socio-economic unrest [100]. However, many refugees are confined to camps and face social exclusion, highlighting the need to integrate them into education. Extensive research has shown that integrating refugee children into education can improve their self-confidence and resilience, enhance community relations, and provide skills necessary for a better future [34,101]. Without formal and non-formal education, however, refugee children risk being marginalized for the long term [102].

To overcome the formal and non-formal education challenges in an emergency situation, the commitment of host nations, donors, practitioners, and other stakeholders are critical [72]. While the GoB has initiated a few pilots formal learning centers, more must be established, and adequate support for quality education must be provided. Collaborative efforts between the host nation, stakeholders, and the refugee community can bring about changes in protection, challenge psychological beliefs and intellectual roles, and prioritize educating refugees during emergencies. Few examples exist of educating refugees during crises with a focus on adverse socialization, exacerbating trauma, escalating conflict, and targeting extremists. Formal and non-formal education can contribute to the development of refugee children, enabling them to apply their knowledge and skills in daily life and promoting sustainable development for a better future.

## 7 Conclusion and recommendations

The crisis in education for the Rohingya, from their displacement in Myanmar to their arrival in Bangladesh, is a complex and long-standing issue that cannot be solved quickly. This crisis was sparked by the decolonisation and democratisation process in Myanmar. Providing formal and non-formal education is critical for the survival of Rohingya children and could be the key to their future success. However, Ullah [103] has argued that the international community has not done enough to bring justice for the Rohingya refugee, while the involvement of the Chinese and India in the region has increased tensions and hindered efforts to promote justice [104].

Education is an essential tool empowering “*learners to make informed decisions and responsible actions for environmental integrity, economic viability, and a just society, for present and future generations, while respecting cultural diversity*” [105]. Education is critical for the future of Rohingya children and political stability and security in Bangladesh. UNESCO [106] also emphasized that education can enhance social and civic competencies including knowledge, skills, and attitudes that promote active citizenship through formal and non-formal education. To achieve educational goals and outcomes, both the teachers and students internalize them. Formal and non-formal education can help students make decisions and prepare them for future livelihoods. However, there is a lack of adequate institutional and structural support for refugee education, and an insufficient number of experienced teachers and logistical challenges have put pressure on the fragile informal education system for refugees in Bangladesh. Addressing the education crisis for refugees requires a focus on formal and non-formal education with long-term targets, specific goals, and funding to produce effective and sustainable outcomes for refugees and host communities.

It is essential to establish a partnership between refugees, donors, and humanitarian agencies to ensure that formal and non-formal education is consistently and sustainably provided through both governmental and non-governmental initiatives. This requires continuous pressure both from within and outside the refugee community, to urge the government of Bangladesh to initiate and support such education programs. Adequate funding, both locally and globally, is also critical for developing curricula, training teachers, and establishing more educational centers. Furthermore, the successful pilot project on formal education must be continued, and quality teaching staff must be recruited and provided with regular training and competitive salaries to ensure the sustainability of education services. Policies and programs for educational certification and community service are also necessary to install confidence among the refugees and promote their integration into society. The importance of formal and non-formal education for refugee children lies in their learning outcomes, which are crucial for their mental well-being. Studies have shown that ‘learning’, through both formal and non-formal education, is a persistent goal for refugee children's education in various locations. As Filippo Grandi, the high commissioner of UNHCR, rightly pointed out, addressing this challenge requires a massive and coordinated effort, which we cannot afford to ignore.

## Author contribution statement

A N M Zakir Hossain: Conceived and designed the experiments; Performed the experiments; Analysed and interpreted the data; Contributed reagents, materials, analysis tools or data; Wrote the paper.

## Data availability statement

Data will be made available on request.

## Additional information

No additional information is available for this paper.

## Declaration of competing interest

The authors declare that they have no known competing financial interests or personal relationships that could have appeared to influence the work reported in this paper.

## References

[1] Crawley H., Setrana M. (2021). Handbook on the Governance and Politics of Migration.

[2] Pascucci E. (2021). More logistics, less aid: humanitarian-business partnerships and sustainability in the refugee camp. World Dev..

[3] Wardeh M., Marques R.C. (2021). Sustainability in refugee camps: a comparison of the two largest refugee camps in the world. J. Refug. Stud..

[4] Momem M.N. (2021). Decolonising Conflicts, Security, Peace, Gender, Environment and Development in the Anthropocene.

[5] Hiitola J. (2021). Family separation and everyday (in) security in the lives of unaccompanied refugee minors. Oñati Socio-Legal Series.

[6] Sanchez Salgado R.M. (2021).

[7] Leider J. (2018). Oxford Research Encyclopaedia of Asian History.

[8] Laoutides C. (2021). Ethnoreligious conflict and populism: emotive political response in the Rohingya conflict. Religions.

[9] Ansar A. (2020). The unfolding of belonging, exclusion, and exile: a reflection on the history of Rohingya refugee crisis in Southeast Asia. J. Muslim Minority Aff..

[10] Shohel M., Mahruf C. (2020). Education in emergencies: challenges of providing education for Rohingya children living in refugee camps in Bangladesh. Education Inquiry.

[11] Dryden-Peterson S. (2016). Refugee education: the crossroads of globalisation. Educ. Res..

[12] Molla T. (2021). Refugee education: homogenised policy provisions and overlooked factors of disadvantage. Int. Stud. Sociol. Educ..

[13] UNHCR (2021). https://reliefweb.int/sites/reliefweb.int/files/resources/612f85d64.pdf.

[14] ISCG (2020). Inter sector coordination group. Education Sector, Cox's Bazar.

[15] BRO (2018). https://burmacampaign.org.uk/media/The-Right-toEducation-Denied-for-Rohingya-%20Refugees-in-Bangladesh.pdf.

[16] BARC (2020). http://www.brac.net/program/wp-%20content/%20uploads/2020/03/Perception-of-People-of-Bangladesh-about-Forcibly-%20DisplacedMyanmar-Nationals-FDMNs.pdf.

[17] Hossain A.Z. (2021). Preparedness for education to Rohingya refugee children in Bangladesh: potentials and challenges to citizenship education. JSSE-Journal of Social Science Education.

[18] Field J., Leicester M. (2003). Lifelong Learning: Education across the Lifespan.

[19] Power C. (2014).

[20] Colardyn D., Bjornavold J. (2004). Validation of formal, non-formal and informal learning: policy and practices in EU member states. Eur. J. Educ..

[21] Cedefop (2014). http://www.cedefop.europa.eu/en/events-and-projects/projects/validation-non-formal-%20and-informal-learning/european-inventory/european-inventory-%20glossary#:%7E:text=Learning%2520that%2520occurs%2520in%2520an,It%2520typical%20ly%2520leads%2520to%2520certification.

[22] Yasunaga M. (2014). https://www.allinschool.org/media/1991/file/Paper-OOSCI-Non-Formal-Education-%20Learning-Needs-2014-en.pdf.pdf.

[23] Debarliev S., Janeska-Iliev A., Stripeikis O., Zupan B. (2022). What can education bring to entrepreneurship? Formal versus non-formal education. J. Small Bus. Manag..

[24] UDHR (1948). Universal declaration of human rights. UN General Assembly.

[25] Okello J., Nakimuli-Mpungu E., Musisi S., Broekaert E., Derluyn I. (2014). The association between attachment and mental health symptoms among school-going adolescents in northern Uganda: the moderating role of war-related trauma. PLoS One.

[26] Lyby E., Crisp J., Talbot C., Cipollone D. (2001). Vocational Training for Refugees: a Case Study from Tanzania.

[27] Hilal R., McGrath S. (2016). The role of vocational education and training in Palestine in addressing inequality and promoting human development. J. Int. Comp. Educ..

[28] Wani M., Phogat P. (2018). Effects on mental well-being of children facing armed conflict: a systemic review. Indian Journal of Health & Wellbeing.

[29] Tubbs Dolan C., Kim H.Y., Brown L., Gjicali K., Borsani S., Houchaimi S.E., Aber J.L. (2022). Supporting Syrian Refugee children's academic and social-emotional learning in national education systems: a cluster randomized controlled trial of nonformal remedial support and mindfulness programs in Lebanon. Am. Educ. Res. J..

[30] Sung J., Wahl R. (2021). Oxford Research Encyclopaedia of Education.

[31] Veck W., Dovigo F., Proyer M. (2021). Editorial: refugees and inclusive education. Int. J. Incl. Educ..

[32] Quintelier E. (2010). The effect of schools on political participation: a multilevel logistic analysis. Res. Pap. Educ..

[33] Stockemer D. (2014). What drives unconventional political participation? A two level study. Soc. Sci. J..

[34] Block K., Cross S., Riggs E., Gibbs L. (2014). Supporting schools to create an inclusive environment for refugee students. Int. J. Incl. Educ..

[35] Chatzidaki A., Tsokalidou R. (2021).

[36] Koehler C., Schneider J. (2019). Young refugees in education: the particular challenges of school systems in Europe. Comparative migration studies.

[37] Prodip M.A., Garnett J. (2019). Comparative Perspectives on Refugee Youth Education.

[38] Cummins J., Tinajero J.V., De Villar R.A. (1999). The Power of Two Languages.

[39] Romi S., Schmida M. (2009). Non‐formal education: a major educational force in the postmodern era. Camb. J. Educ..

[40] Kahane R. (1997).

[41] Cohen E.H. (2001). A structural analysis of the R. Kahane code of informality: elements toward a theory of informal education. Socio. Inq..

[42] United Nations (1948). https://www.un.org/en/universal-declaration-human-rights/.

[43] United Nations (1989). https://www.ohchr.org/en/professionalinterest/pages/crc.aspx.

[44] Gay G. (2010).

[45] Lantolf J.P. (2000).

[46] Becker B., Raschke E., Vieluf S., Böse S., Laschewski A., Rauch D., Stošić P. (2023). Teaching refugee students: the role of teachers' attitudes towards cultural diversity. Teachers and Teaching.

[47] Cerna L. (2019).

[48] Pappas C., Williams I. (2011). Grey literature: its emerging importance. J. Hosp. Librarian..

[49] Jesson J., Matheson L., Lacey F.M. (2011).

[50] Aung S.M.T. (2016).

[51] Hossain A.N.M. (2020). Rohingya refugee and resettlement nexus in Bangladesh: why it become a research agenda?. Journal of Social and Political Sciences.

[52] Johnson H.G., Johnson Harry G. (1967). Economic Nationalism in Old and New States.

[53] Bessel Richard, Claudia Bettina Haake (2009).

[54] Stone Dan (2018). Refugees then and now: memory, history and politics in the long twentieth century: an introduction. Patterns Prejudice.

[55] Fair C., Christine (2018). Rohingya: victims of a great game east. Wash. Q..

[56] Taylor Robert H. (2009).

[57] Gemie Sharif, Humbert Laure, Reid Fiona (2012).

[58] Cohen Albert (1949). *The Aims of the International Refugee Organization As Regards Legal and Political Protection*, Speech at IRO and Voluntary Organizations Conference.

[59] BenEzer Gadi, Zetter Roger (2015). Searching for directions: conceptual and methodological challenges in researching refugee journeys. J. Refug. Stud..

[60] Kushner Antony Robin Jeremy (2017).

[61] Lintner Bertil (2015).

[62] Murshid Navine (2018). Bangladesh copes with the Rohingya crisis by itself. Curr. Hist..

[63] Gatrell Peter (2013).

[64] UNESCO (2005). Guidelines for inclusion: ensuring access to education for all. http://unesdoc.unesco.org/images/0014/001402/140224e.pdf.

[65] UNHCR (2020). Joint Government of Bangladesh - UNHCR Population factsheet as of October 31 2020. https://data2.unhcr.org/en/documents/details/82872.

[66] Hamilton Richard, Moore Dennis (2003).

[67] Eichler S. (2019). http://urn.kb.se/resolve?urn=urn:nbn:se:hj:diva-%2047238.

[68] The Daily Star (2018). Entire generation denied education. The Daily Star. https://www.thedailystar.net/backpage/news/entire-%20generation-denied-education-1673896.

[69] Farzana Kazi Fahmida (2016). Voices of the Burmese Rohingya refugees: everyday politics of survival in refugee camps in Bangladesh. Journal of Social Science and Humanities.

[70] Human Rights Watch (2019). Are we not human?. Denial of Education for Rohingya Refugee Children in Bangladesh.

[71] ISCG (2021). https://reliefweb.int/sites/reliefweb.int/files/resources/dasboard_cxbes_jan_21_ma_v3.p%20df.

[72] GCR (2018). Report of the United Nations High Commissioner for Refugees 2018, Part II.

[73] Appleby J.K. (2020). Implementation of the global Compact on safe, orderly, and regular migration: a whole-of-society approach. Journal on Migration and Human Security.

[74] Goodwin-Gill G.S. (2018). The global compacts and the future of refugee and migrant protection in the Asia Pacific Region. Int. J. Refug. Law.

[75] REACH (2021). https://www.impact-repository.org/document/reach/1a8e426c/REACH_Education-Sector-%20Assessment_Thematic-Briefs_Msluarch_2021.pdf.

[76] UNHCR (2021). https://reliefweb.int/sites/reliefweb.int/files/resources/612f85d64.pdf.

[77] Coombes Andi, Ponta Oriana (2019). Teachers in Crisis Contexts: Promising Practices in Teacher Management.

[78] UIS (2012). http://uis.unesco.org/sites/default/files/documents/international-standard-classification-of-education-isced-2011-en.pdf.

[79] Education Sector Dashboard (2018). Education sector 4W analysis. https://reliefweb.int/report/bangladesh/education-sector-%20dashboard-coxs-bazar-bangladesh-12-august-2018.

[80] Karim Azmina, Hussain Faheem (2019). International Conference on Social Implications of Computers in Developing Countries.

[81] Sector Education (2018). Joint education needs assessment: Rohingya Refugee in Cox's Bazar. https://reliefweb.int//reportbangladesh/joint-education-needs-assessment-rohingya-%20refugee-%20cox-s-bazar-june-2018.

[82] Pranay Sharma (2018). Balancing the wheel: with polls pending, khaleda zia in jail and hasina pampering islamists. Bangladesh Is on Edge, Outlook.

[83] Altinyelken H.K. (2021). Critical thinking and non-formal Islamic education: perspectives from young Muslims in The Netherlands. Contemporary Islam.

[84] Md Zahed I.U., Prodip M.A. (2021).

[85] Hassan Mohammad Mehedy, Walker Katherine, Rahman Munshi Khaledur, Southworth Jane, Audrey Culver Smith (2018). Rohingya refugee crisis and forest cover change in Teknaf, Bangladesh. Rem. Sens..

[86] NRC (2009). https://www.nrc.no/news/2019/august2/ngos-warn-of-worsening-crisis-in-myanmar-call-%20for-refugees-engagement-on-safe-voluntary-returns/.

[87] The Daily Star (2020). Bangladesh allows education for Rohingya children. The Daily Star. https://www.thedailystar.net/rohingya-%20crisis/news/bangladesh-allows-education-rohingya-refugee-children-1860280.

[88] UNHCR (2020). https://www.unhcr.org/news/press/2020/3/5e5cfb984/un-appeals-us877-million-rohingya-refugee-response-bangladesh.html.

[89] Inter-Agency Network for Education in Emergencies (INEE) (2010).

[90] Miller J., Mitchell J., Brown J. (2005). African refugees with interrupted schooling in the high school mainstream: dilemmas for teachers. Prospect.

[91] Das H.K., Shafiq M.S., Islam G.M.R. (2021). Formulation of teacher's competency framework in the context of Rohingya refugee education in Bangladesh: lessons learned. Refugee education in South Asia: Policies and perspectives.

[92] Sutton D., Kearney A., Ashton K. (2021). Improving educational inclusion for refugee-background learners through appreciation of diversity. Int. J. Incl. Educ..

[93] Maber E.J. (2016). Cross-border transitions: navigating conflict and political change through community education practices in Myanmar and the Thai border. Glob. Soc. Educ..

[94] Dryden-Peterson S. (2017). Refugee education: education for an unknowable future. Curric. Inq..

[95] Hayward M. (2017). Teaching as a primary therapeutic intervention for learners from refugee backgrounds. Intercult. Educ..

[96] Bakali N., Wasty S. (2020). Identity, social mobility, and trauma: post-conflict educational realities for survivors of the Rohingya genocide. Religions.

[97] David-Erb M. (2021). Language of instruction: concerning its choice and social prestige in Burkina Faso. Int. Rev. Educ..

[98] Suma J.T. (2022). Global-Local Tradeoffs, Order-Disorder Consequences: 'State'No More an Island?.

[99] Suma J.T. (2022). Rohingya Camp Narratives: Tales From The ‘Lesser Roads’ Traveled.

[100] Brown A., Roche J., Hurley M. (2020). Engaging migrant and refugee communities in non-formal science learning spaces. Journal of Science Communication.

[101] Slee R. (2011).

[102] Farzana K.F., Pero S.D.M., Othman M.F. (2020). The dream's door: educational marginalization of Rohingya children in Malaysia. South Asian Journal of Business and Management Cases.

[103] Ullah A.K., Ahsan M. (2016). Rohingya crisis in Myanmar: seeking justice for the "stateless. J. Contemp. Crim. Justice.

[104] Rashid S.R. (2020). Finding a durable solution to Bangladesh's Rohingya refugee problem: policies, prospects and politics. Asian Journal of Comparative Politics.

[105] United Nations Educational (2018). https://en.unesco.org/themes/education-sustainable-development/what-is-esd.

[106] UNESCO (2011). https://inee.org/system/files/resources/212715eng.pdf.

